# Supplementary material for: Proteomic profiling of drug and nutrient transporter expression by small intestinal region in neonates, pediatrics, and adults
Source: bioRxiv. 2026 Jun 17:2026.06.13.731200. Preprint. [Version 1] doi: 10.64898/2026.06.13.731200 (PMC13307956; doi:10.64898/2026.06.13.731200)
Supplement: Supplement 1 [file media-1.pdf]

Proteomic profiling of drug and nutrient transporter expression by small intestinal region in neonates, pediatrics, and adults

Vicky L. Sun, Kai Wang, Edward J. Kelly, Samuel L.M. Arnold*
*corresponding author

Methods

Small intestine dissection

Duodenum was identified by the presence of adjacent pancreatic tissue (if present), circular folds, a wider diameter relative to the jejunum or ileum, and the duodenojejunal flexure (if present). Jejunum was identified by a smaller diameter relative to the duodenum and the presence of circular folds that were at higher density than in the duodenum. Ileum was identified by the presence of a smaller diameter relative to the jejunum, sparse circular folds, the ileocecal junction (if present), and the presence of adjacent cecum/appendix tissue (if present).

Donor exclusion criteria included hepatitis B (antigen, core, NAT) positive serology, hepatitis C positive serology, HIV positive serology, bacterial or viral meningitis, MRSA, RPR/syphilis positive serology, sepsis, and approximate cold ischemic time > 24 hours. Exclusion criteria were implemented prior to sample collection.

Kingfisher sample preparation protocol

Reagent info

Plate	Contents	Well volume (µL)
1-Protein Capture	100% Acetonitrile	520
	Lysate	210.3
	Beads	12.5
2-ACN Wash 1	95% Acetonitrile	1000
3-ACN Wash 2	95% Acetonitrile	1000
4-ACN Wash 3	95% Acetonitrile	1000
5-EtOH Wash 1	70% Ethanol	1000
6-EtOH Wash 2	70% Ethanol	1000
7-Trypsin Elution	50 mM ABC, Trypsin 1:20	50
Tip Plate	n/a	n/a

Steps Data

Step	Plate	Details
Pick-Up	Tip Plate	n/a
Bind Proteins	P1-Protein Capture	
Beginning of step	Precollect	No
Mixing/heating	Shake 1 time, speed	00:01:00, Medium
	Shake 2 time, speed	00:10:00, Paused
	Loop Count	2
	Tip position when paused	Tip edge in liquid
	Heating during mixing	No
End of step	Postmix	No
	Collect count	5

	Collect time (s)	30
Wash 1	P2-ACN Wash 1	
Beginning of step	Precollect	No
	Release beads	No
Mixing/heating	Mixing time, speed	00:02:30, Slow
	Heating during mixing	No
End of step	Postmix	No
	Collect beads	No
Wash 2	P3-ACN Wash 2	
Beginning of step	Precollect	No
	Release beads	No
Mixing/heating	Mixing time, speed	00:02:30, Slow
	Heating during mixing	No
End of step	Postmix	No
	Collect beads	No
Wash 3	P4-ACN Wash 3	
Beginning of step	Precollect	No
	Release beads	No
Mixing/heating	Mixing time, speed	00:02:30, Slow
	Heating during mixing	No
End of step	Postmix	No
	Collect beads	No
Wash 4	P5-EtOH Wash 1	
Beginning of step	Precollect	No
	Release beads	No
Mixing/heating	Mixing time, speed	00:02:30, Slow
	Heating during mixing	No
End of step	Postmix	No
	Collect beads	No
Wash 5	P6-EtOH Wash 2	
Beginning of step	Precollect	No
	Release beads	No
Mixing/heating	Mixing time, speed	00:02:30, Slow
	Heating during mixing	No
End of step	Postmix	No
	Collect beads	No
Pause	P7-Trypsin Elution	
	Message	Add digestion/elution plate
Protein Digestion and Elution	P7-Trypsin Elution	
Beginning of step	Precollect	No
	Release time, speed	00:00:20, Bottom mix
Mixing/heating	Shake 1 time, speed	00:00:15, Medium
	Shake 2 time, speed	00:02:15, Paused
	Loop Count	24
	Tip position when paused	Tip edge in liquid

	Heating temperature (˚C)	37
	Preheat	Yes
End of step	Postmix	No
	Collect count	5
	Collect time (s)	30
Dispose Beads	P1-Protein Capture	
	Release time, speed	00:00:30, Medium
Leave	Tip Plate	

Matched-matrix calibration curve

Yeast (Saccharomyces cerevisiae, Type II (Baker's yeast), Sigma Aldrich, St. Louis, MO) was lysed by sonication (Fisher Scientific sonic dismembrator model 100, set to continuous at 4 or 5, 10-second intervals for 1 minute) in 500 µL 20 mM Tris, 50 mM mannitol, 1 mM EDTA buffer at pH 7.4 (adjusted with HCl) with protease inhibitor. Protein concentration in yeast lysate was estimated with the BCA assay and aliquots of yeast lysate were digested as described in the Methods (Sample preparation and Protein digestion sections).

Standards were prepared by diluting digested, pooled mucosa samples (after sample reconstitution, described in Methods under Peptide sample analysis by DIA-MS) in digested yeast lysate. The following standards (expressed as % of pure pooled mucosa) were prepared in each calibration curve: 0.5, 1, 5, 10, 25, 50, 70, and 100%. Each standard had a total peptide concentration of 0.5 µg/µL in the reconstitution solution below. Calibration curves were prepared in technical triplicate and each curve was injected from low to high mucosa percentage once on the mass spectrometer.

Chromatogram library generation

Equal volumes of digested mucosa samples and 1 µL of digested yeast enolase were combined to generate a chromatogram library sample. A chromatogram library was generated by gas-phase fractionation of 1 µg of library sample on-column with narrow-window DIA runs of 4 m/z windows (with overlapping by 2 m/z)[30]. Each gas-phase fraction spanned 100 m/z with a total of 6 fractions spanning 395-1005 m/z. Narrow-window DIA runs were acquired (25 second expected precursor peak width and +3 charge, ion accumulation time of 23 ms, positive polarity, NCE of 27, 30,000 resolution, automatic gain control target of 1,000,000) with two MS scans to acquire precursor spectra every 25 MS/MS spectra. One MS scan covered the target m/z range ± 5 m/z (e.g., 395-505 m/z) while the other MS scan spanned 400-1600 m/z. Both MS scans had an expected precursor peak width of 25 seconds and charge of 3, ion accumulation time of 23 ms, positive polarity, 60,000 resolution, and an automatic gain control target of 1,000,000.

Quantitative peptide identification

Quantitative peptides were identified for all small intestine marker proteins, SLC/ABC transporters highlighted in dynamic range plots, and SLC/ABC transporters that were differentially expressed (see Results). Quantitative peptides for these select proteins were identified by examining a bi-linear regression fit to the matched-matrix calibration curves in Skyline.

Supplemental Figures and Tables

Table S1. Donor demographics and sample information.

Sample Identifier	Sex	Age	Race	Cause of death	Notes
HINT 2	F	5 days old	White (Hispanic)	Anoxia, Cardiovascular (maternal uterine rupture)	n/a
HID04	M	1 month old	White	Anoxia, Cardiovascular (shoulder dystocia)	Only jejunum received
PHI4	M	1 month old	Black or African American	Anoxia, Cardiovascular (aspiration)	Only duodenum and jejunum received
PHI5	M	12 months old	White	Head Trauma (MVA)	n/a
HINT 3	M	2 years old	White	Unknown (natural causes)	n/a
PHI2	F	2 years old	not reported	Anoxia, Cardiovascular (non-MVA)	Only ileum analyzed
PHI3	M	4 years old	White	Head Trauma (MVA)	n/a
HINT 1	M	5 years old	African American	Drug-induced toxicity	n/a
PHI6	M	5 years old	White	Anoxia (drowning)	n/a
PHI1	F	8 years old	Black or African American	Anoxia, Cardiovascular (cardiac)	Only jejunum and ileum received
AHI7	M	18 years old	White	Anoxia (marijuana/opioid intoxication)	n/a
AHI2	M	20 years old	White	Anoxia, Cardiovascular (natural causes)	Only jejunum received
AHI8	F	23 years old	White	Anoxia (tylenol intoxication)	n/a
AHI4	F	24 years old	White	Cerebrovascular/stroke (intracranial hemorrhage/stroke)	Only ileum received
AHI6	F	27 years old	White	Anoxia, Cardiovascular	n/a
AHI5	F	32 years old	White	CNS tumor	Only jejunum and ileum received
AHI1	M	34 years old	African American	Anoxia, Cardiovascular (natural causes)	Only jejunum and ileum received; donor had diabetes
AHI3	M	34 years old	White	Anoxia, Cardiovascular (natural causes)	Only jejunum and ileum received; donor had pancreatitis

F: female, M: male, MVA: motor-vehicle accident, n/a: not applicable

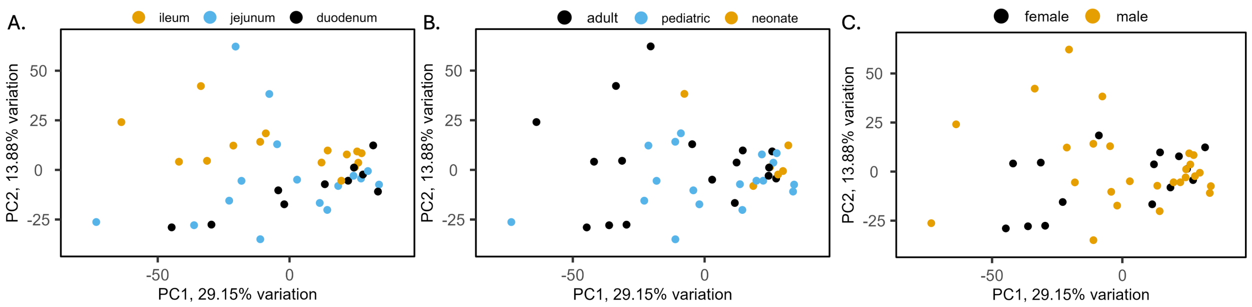

Figure S1. PCA of samples by intestinal region, age, or sex. Samples cluster by proximal small intestine regions (duodenum or jejunum) or ileum (A) but not strongly by age group (B). No clustering by donor sex was observed (C). PC = principal component

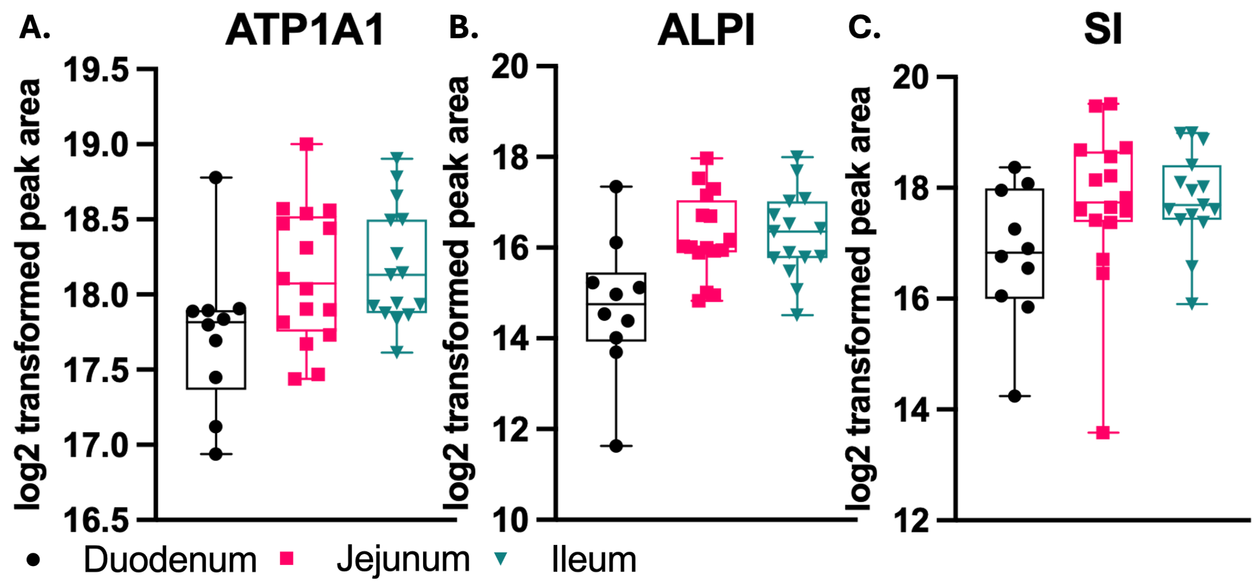

Figure S2. Small intestine marker proteins by intestinal region. The small intestine marker proteins ATP1A1 (A), ALPI (B), and SI (C) did not show trends with small intestine region.

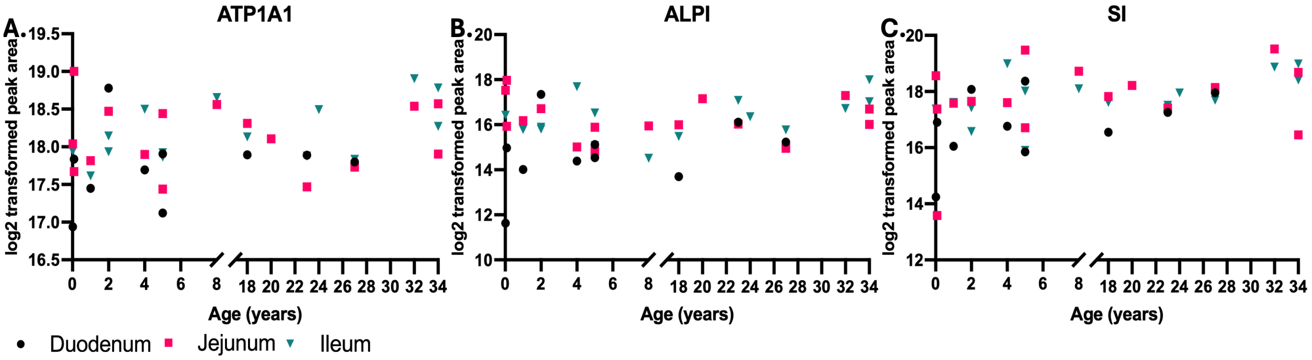

Figure S3. Small intestine marker proteins by age. ATP1A1 (A), ALPI (B), and SI (C) did not have a significant trends by small intestinal region with age.

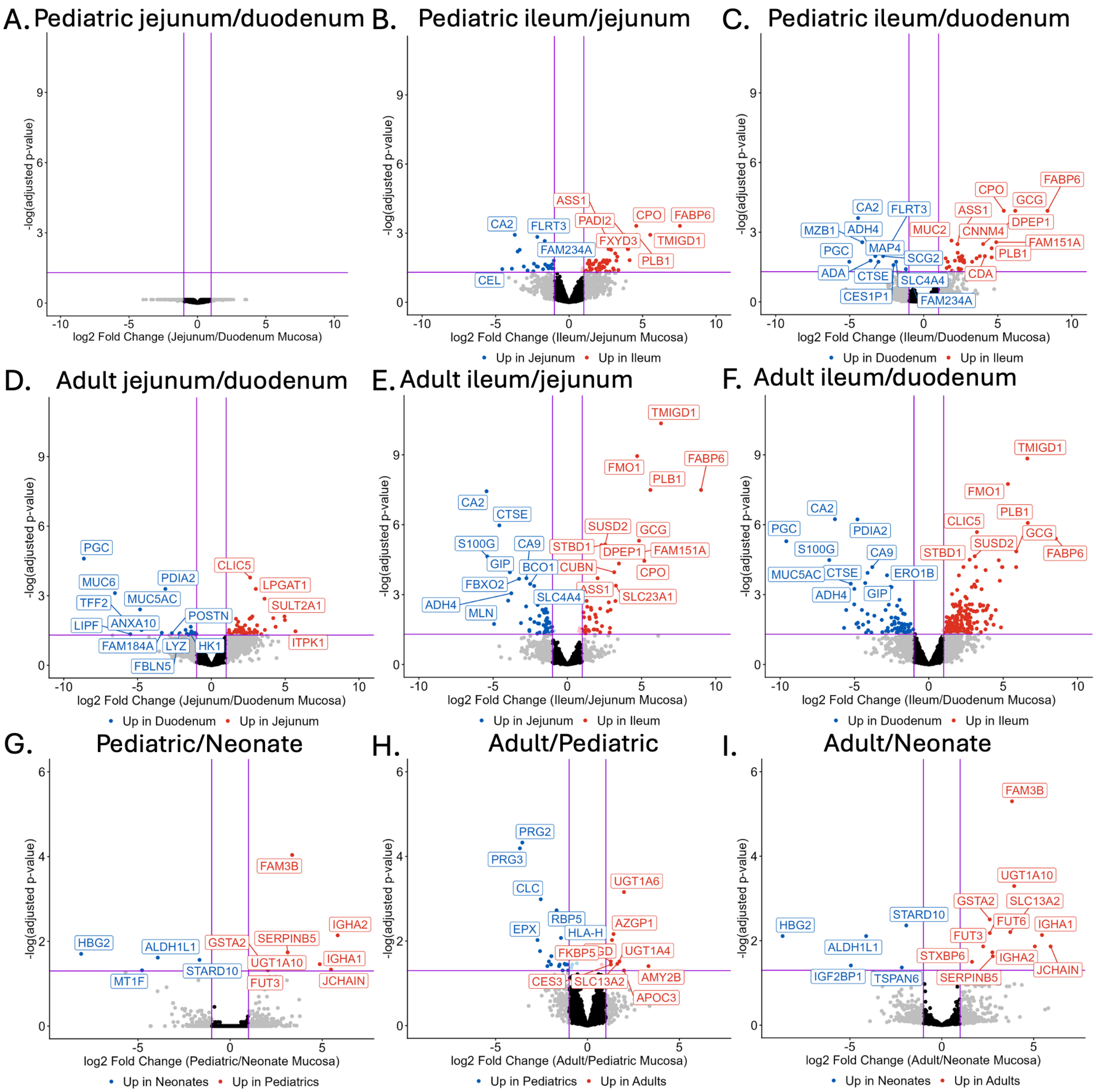

Figure S4. Differentially expressed proteins by intestinal region or age. Pediatric regions (A-C), adult regions (D-F), age groups (G-I). No SLC or ABC transporters are differentially expressed between neonates and pediatrics (G), but SLC13A2 is significantly higher with age in adults compared to pediatrics (H) or neonates (I). n = 5 pediatric duodenum, n = 6 pediatric jejunum, n = 7 pediatric ileum, n = 3 adult duodenum, n = 7 adult jejunum, n = 7 adult ileum; t-tests with multiple testing corrected by Benjamini-Hochberg method; pediatric duodenum versus jejunum n = 5,762 proteins, pediatric jejunum versus ileum n = 5,784 proteins, pediatric duodenum versus ileum n = 5,759 proteins, adult duodenum versus jejunum n = 5,657 proteins, pediatric jejunum versus ileum n = 5,771 proteins, pediatric duodenum versus ileum n = 5,648 proteins, pediatric versus neonate n = 5,725 proteins, adult versus pediatric n = 5,867 proteins, adult versus neonate n = 5,725 proteins
